# Isoform-specific vs. pan-histone deacetylase inhibition as approaches for countering glioblastoma: an *in vitro* study

**DOI:** 10.3389/fonc.2025.1695552

**Published:** 2025-11-27

**Authors:** Ameya Joshi, Natasha Ratnapradipa, Jayce Hughes, Erik Moore, Andrew Ekpenyong, Surabhi Shukla

**Affiliations:** 1 Pharmacy Sciences Department, School of Pharmacy and Health Professions, Creighton University, Omaha, NE, United States; 2 Physics Department, Creighton University, Omaha, NE, United States

**Keywords:** glioblastoma, HDAC inhibitors, Vorinostat, Trichostatin-A, Tubacin, temozolomide

## Abstract

**Introduction:**

Glioblastoma (GBM) is a grade 4 brain tumor that originates in astrocytes. GBM has a high death rate and a very low median patient survival rate of 14–16 months, even with well-established treatment regimens. The majority of preclinical models assess the effectiveness of molecular leads on two-dimensional (2D) cell cultures, which may provide insight into toxicity against particular genotypes of GBM but do not provide insight into the mechanism of action of the therapeutic drug. Three-dimensional (3D) cultures present an attractive alternative due to their ability to closely model in vivo tumor-like conditions.

**Methods:**

In the present study, we used a rotary cell culture technique to culture 3D cancer spheroids of the T98G cell line. Initially, we estimated the relative potency of histone deacetylase (HDAC) inhibitors, which are molecular leads currently in clinical trials as epigenetic therapy for GBM, on 2D and 3D cultures of T98G. We characterized the effect of the 3D half-maximal inhibitory concentrations (IC50) on spheroids using a live–dead assay to figure out which inhibitors inhibited cell viability in 3D the most. Finally, we checked the effects of the non-specific and specific inhibitors on tumor migration dynamics using an electric cell impedance sensing (ECIS) device with the help of two parameters—rate of migration (ROM) and late resistance (LR).

**Results and discussion:**

Our results show that the specific HDAC-6 inhibitor Tubacin had a more potent anti-proliferative effect in both the cytotoxicity and live–dead assays. The non-specific inhibitor Vorinostat surprisingly promoted migration in the cells at its 2D IC50 value treatment, and none of the inhibitors was able to significantly decrease late resistance compared to untreated controls, indicating the need for the development of more potent HDAC inhibitors for monotherapy for GBM.

## 1 Introduction

Glioblastoma (GBM) is a grade 4 tumor originating mainly in astrocytes. It comprises up to 48% of total CNS tumor cases (1). GBM primarily affects adults age 60 and above and has a region non-specific higher incidence in male patients (2). It originates from mutations in genes like phosphatase and tensin homolog (PTEN), isocitrate dehydrogenase 1/2 (IDH 1/2), tumor protein p53 (TP53), mouse double minute 2/4 (MDM 2/4), and epidermal growth factor receptor (EGFR), among others, which molecularly lead to a cascade of dysregulatory metabolic changes like altered substrate affinity to α-ketoglutarate (3), its conversion to harmful metabolites like 2-hydroxglutasrate (4), and the latter’s harmful downstream effects ranging from blockading dioxygenases to hypoxia-inducible factor 1-alpha (HIF-α) activation (3–5).

Current treatment options involve surgical removal via craniotomy, followed by radiation therapy and chemotherapy (6). Craniotomy involves surgical removal of bulk tumor mass with subsequent integrated radio-chemotherapy used as a subsequent measure to kill remaining tumor cells (7). External beam radiation therapy remains the most common radiotherapy approach (8), though alternatives like internal radiation (with Cobalt-60 and Cesium-137), proton beam therapy (PBT), and gamma knife radiosurgery have shown increasing promise (9). For chemotherapy, Temozolomide (TMZ) is considered as the gold standard drug for GBM and is the first-line chemotherapy for newly diagnosed cases (10). During advanced stages, it is sometimes assisted with drugs like Lomustine and Bevacizumab. Despite standardized multimodal treatment plans in place, patients with GBM still have a high mortality rate, with a median survival of 1 to 1 1/2 years post prognosis (11). This highlights a need for discovering newer prospective molecular leads for reducing proliferation of GBM.

Most preclinical studies for screening new molecular leads for GBM largely relies on testing on monolayer cultures of cell lines like T-98G, U25MG, A172, LN-229, C6, and U87MG, among others (12). Although two-dimensional (2D) assays are straightforward, high-throughput, and give an idea of cytotoxicity of the agents against specific GBM genotypes, they neglect essential elements of *in vivo* gliomas like cell–cell interactions, extracellular matrix interactions, hypoxic/necrotic gradients, and phenotypic heterogeneity. This collectively contributes to discrepancies between *in vitro* efficacy and *in vivo* or clinical trial outcomes (13).

Three-dimensional (3D) cell cultures present an attractive alternative to monolayer cultures due to greater complexity in cytoarchitecture, closer recapitulation of tumor microenvironments, and biomarker expression that more closely mimics *in vivo* conditions (14, 15). It is observed that 3D cultures have higher resistance to drug concentrations, compared with monolayer cultures (16). Among 3D models, neurospheres are not only the easiest to develop, but also the most limited models. Cancer spheroids are characterized by the presence of a necrotic core surrounded by proliferative peripheral layers. Organoids, 3D bioprinting, tissue slice cultures, and tumor-on-chip are more advanced 3D cell culture models that can house multiple cell types and record larger diameters and high marker heterogenicity. However, higher models come with more constraints liked cost, complexity, limited scalability for screening purposes, or standardization challenges (17, 18). Consequently, there is a persistent need for an intermediate 3D tumor model platform that is reproducible, scalable, and amenable to drug screening.

**Figure 1:**
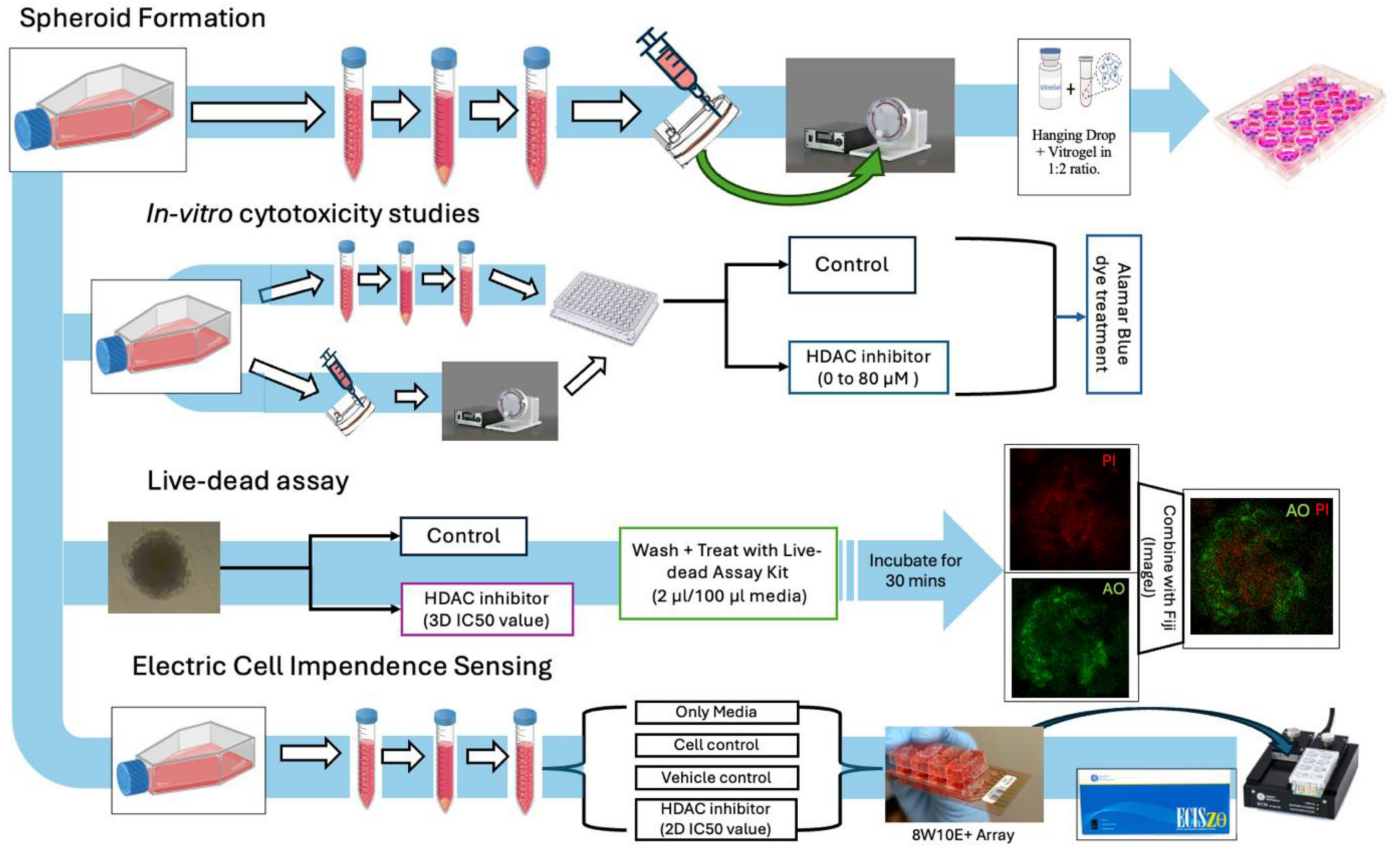
Study overview: general workflow of assay planning from spheroid generation, *in vitro* cytotoxicity studies, live–dead assays, and migration studies.

In the current study, we present a simulated microgravity-based spheroid formation pipeline combined with ECM-embedded culture and functional assays (see **Figure 1**). Simulated microgravity was a technology initially developed by NASA to study the impact of microgravity on physiological functions (19–21) and to develop artificial organs (22–25). Microgravity systems later emerged as a method to make 3D spheroids for *in vitro* studies in both cancer cells and stem cells (26, 27). Multiple cancer cell lines, both adherent and suspension, have been used to generate spheroids to model respective cancers (28–33). Our initial goal was to generate spheroids using a Rotary Cell Culture System (RCCS) via an in-house spheroid generation protocol (32) and then transfer them on an ECM-embedded culture and, finally, to test molecular leads against them.

We compared the effects of three histone deacetylase (HDAC) inhibitors, Vorinostat (a pan-HDAC inhibitor), Trichostatin A (a broad-spectrum HDAC inhibitor), and Tubacin (an HDAC isoform-6 specific inhibitor), on the 3D spheroids using Alamar Blue and live–dead assays. HDACs are enzymes that cleave acetyl groups from both histones and non-histone targets, and majorly regulate gene expression and regulation (34). Their dysregulation is implicated in various malignancies including GBM (35).

The very first described HDAC inhibitors such as Vorinostat, Romidepsin, Belinostat, or Panobinostat are “first generation” inhibitors and are non-selective in their inhibitory action (36). Many of them are FDA approved for various malignancies (37–40). Vorinostat (SAHA) and Trichostatin A are currently under preclinical evaluation for GBM (41). They are being explored as both stand-alone therapies and synergistic agents to sensitize cancer cells to radiotherapy, immunotherapy, virotherapy, and other chemotherapeutic agents like BR/topoisomerase I inhibition. Vorinostat is currently under Phase II clinical trials as a synergistic agent to TMZ, Bevacizumab, Isotretinoin, Erlotinib, Bortezomib, and Irinotecan (42). Trichostatin A has shown positive results in previous *in vitro* studies as stand-alone treatment (43) and has demonstrated some synergy with TMZ (44).

Specific HDAC inhibition is a more recent approach being studied to treat cancers and other diseases. Specific or second-generation inhibitors selectively inhibit either a particular class or a particular isoform of HDACs within the cells (45). The rationale behind using class-specific or isoform specific inhibitors instead of pan-inhibitors is to potentially reduce adverse effects like myelosuppression (reduced bone marrow activity) (46), cardiotoxicity (in terms of QT prolongation or ventricular arrythmia) (47), or immunosuppression (48). That being said, pan-inhibitors can be beneficial in shutting down multiple tumor survival genes and pathways (49). As a result, there is no clear conscience on whether specific or pan-HDAC inhibitors are better monotherapies or synergistic agents. Specific HDAC isoform-6-specific inhibition as a synergistic approach for GBM has been previously tested on monolayer agar colony formation assays in GBM cell lines and PTEN tumors in general (50, 51). We evaluated the HDAC-6 isoform-specific inhibitor Tubacin alongside SAHA and Trichostatin A, to compare the relative potency of anti-cancer effects of specific inhibitors with pan-inhibitors.

We used a standard resazurin assay to evaluate the relative potency of the three HDAC inhibitors on monolayer and spheroidal cultures of T98G. The half-maximal inhibitory concentrations of the inhibitors on the cultures were computed using non-linear regression. The IC_50_ values for 3D cultures obtained from respective HDAC inhibitors were then used to treat cancer spheroids freshly transferred into ECM-embedded cultures. We developed a live–dead imaging technique to measure viability changes of cells on the cancer spheroids stained with acridine orange-propidium iodide (AO-PI). Spheroid viability was measured as a function of ratio of staining intensity by the live cell dye (AO) to that of the necrotic cell dye (PI). We found that the specific HDAC-6 inhibitor Tubacin had lower IC_50_ values compared to the pan-inhibitors Vorinostat and Trichostatin A in both monolayer and spheroidal cultures (though more significantly in the latter). Tubacin also had significantly lower spheroid viability recorded during the live–dead imaging time points; however, the three HDAC inhibitors showed variant viability changes.

The effect of these HDAC inhibitors on tumor cell migration was checked using an electric cell impedance sensing (ECIS) device. ECIS is a non-invasive technique used for characterizing changes in cellular characteristics over a long time interval (52). ECIS records changes in resistance, impedance, and conductance at the base of electrodes in specialized 8-well arrays in which cancer cells with/without treatments are plated. These changes are typically recorded for the period of 1 week or 168 h to evaluate the long-term effects of treatments. The changes in resistance are correlated with the attachment of cells at the base of the electrode, their proliferation and monolayer formation, and finally metastasis-like changes towards the end (53).

We have previously used this technique to characterize effects of first-line GBM treatments like TMZ and Durvalumab on migration and metastasis on T98G cells (54). In the present study, ECIS was used to determine the effects of stand-alone treatment of the three HDAC inhibitors on migration and metastasis. Two main parameters were measured, which are the “rate of migration” (ROM) and “late resistance” (LR). ROM is the apparent rate at which freshly plated tumor cells (in each condition) migrate at the base of the ECIS microarray electrode and attached to grow in the initial phase of the experiment. This phase is easily visualizable for adherent cell lines like T-98G as resistance almost linearly increases with time before it plateaus (indicating complete monolayer formation). LR gives an idea about cell survival under various treatment conditions, towards the end of the experiment, after the migratory phase. It is a single value that takes into account the summation of resistances post-migratory phase and normalizes it to time and initial resistance per microarray well. We found that Vorinostat, the most-used HDAC inhibitor currently in clinical trials for GBM (42), at its 2D IC_50_ concentration (13.43 µM) increased migration and proliferation in the beginning phase as well as towards the end of the 168-h study. The other HDAC inhibitors, Trichostatin A and Tubacin, also were not able to significantly reduce ROM and LR, indicating potential inadequacies as stand-alone therapies. These negative/mixed results underscore that even HDAC6-specific inhibition may not be sufficient on its own to prevent or prophylactically reduce metastatic effects of migratory GBM cells.

## 2 Materials and methods

A rough overview of methodologies presented in the study is depicted in **Figure 1**.

### 2.1 Cell culture

The T98G cell line was ordered from ATCC (Manassas, VA) and cultured in Dulbecco’s Modified Eagle Medium (DMEM) containing 10% fetal bovine serum (FBS) and 1% penicillin/streptomycin (P/S) stock (ordered from ATCC). Cells were cultured at 37°C, 95% humidity, and 5% CO_2_.

### 2.2 Rotary cell culture system

**Figure 2:**
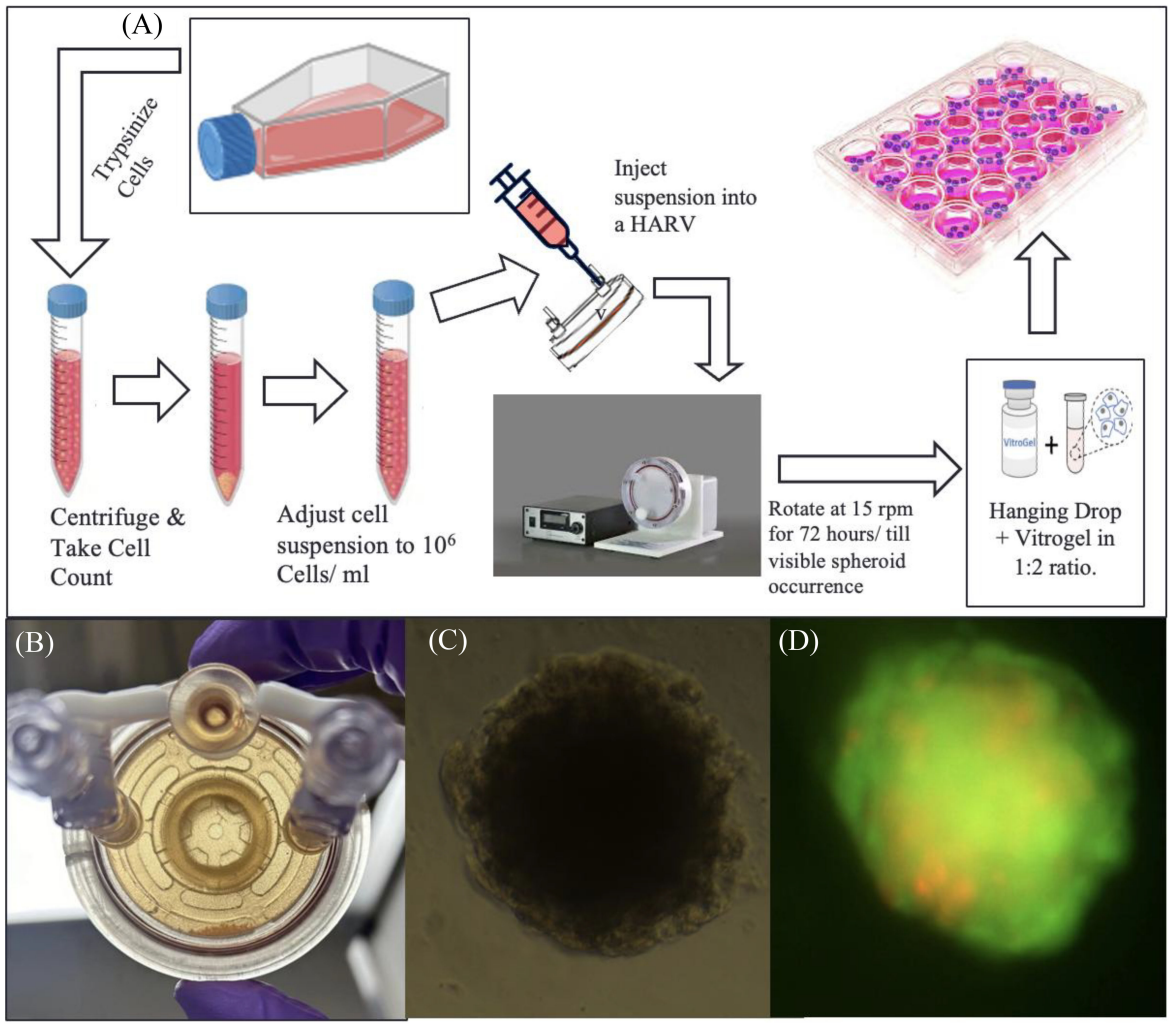
RCCS culture representation. **(A)** Flowchart of spheroid generation method. **(B)** Image of a high-aspect rotating vessel (HARV) containing spheroids, which sink to the bottom part of the vessel when taken out of the rotating system. **(C)** Brightfield image of T98G spheroid transferred to a 96-well plate after encapsulation in Vitrogel hydrogel solution. **(D)** Fluorescence image of the transferred spheroid stained with acridine orange–propidium iodide, for live–dead assay.

**Figure 2A** represents the general workflow to set up RCCS culture and extract spheroids from it. Under aseptic conditions, T-98G cells were lifted from their flasks using Trypsin-EDTA reagent, counted using Trypan Blue Exclusion on a Countess III Cell Counter (Invitrogen, Waltham, MA), and a highly concentrated cell suspension of 10^6^ cells/mL (in DMEM) was prepared. Ten to twelve milliliters of this suspension is required per experiment, along with a 10-mL syringe and a 10-mL High Aspect Rotating Vessel or “HARV” ordered from Synthecon (Houston, TX).

The suspension is slowly drawn into the HARV via one of its syringe ports, using the 10-mL syringe. The HARV was then placed inside a rotator within a CO_2_ incubator and rotated at 15 rpm for a minimum of 72 h or until spheroids are visible (**Figure 2B**). Media from the HARV changed at a 75% media change rate every other day. For changing media, the rotation of the HARV is paused and the spheroids are allowed to settle at the bottom. The supernatant media is then changed.

The spheroids were transferred via encapsulation of cell suspension with the Vitrogel hydrogel solution from TheWell Bioscience (North Brunswick, NJ) in a 1:2 ratio and 50 µL of the mixture was plated onto each well of a flat-bottom 96-well plate and allowed to set at 37°C for 15 min (for soft gel formation) **Figure 2C**. This was followed by the addition of 50 µL of additional DMEM media on top of the set gel. Media was changed from this setup at 60% change rate daily. (The soft gel should not be disturbed; thus, avoid using Pasteur’s pipette–pump assembly for aspiration, as this can suck up the soft gel along with the encapsulated spheroid). **Figure 2D**, shows fluorescent image of transferred spheroid stained with acridine-orange- propidium.

### 2.3 Alamar Blue cytotoxicity assay

A cytotoxicity assay was performed using 54 out of 96 wells on a microplate. T98G cells were seeded at a density of 3,000 cells/well. After overnight incubation, the cells were treated with either media or working solutions of Vorinostat (Selleckchem, Houston, TX), Trichostatin A (Selleckchem, Houston, TX), or Tubacin (Ambeed, Buffalo Grove, II) in DMEM in concentration ranges of 0–80 µM. Each treatment concentration was repeated in triplicate (*n* = 3). After 48 h, media was changed in all wells, along with the addition of fresh media and 10 µL of Alamar Blue HS viability reagent (InVitrogen A50100, Waltham, MA). After 4 h of incubation, fluorescence was measured at an absorption wavelength of 570 nm and an emission wavelength of 600 nm, using a Synergy H1 Hybrid Plate Reader (BioTek, Winooski, VT).

The cytotoxicity assay was also performed on the spheroid cultures plated in flat-bottom 96-well plates. After transferring the spheroids from the rotating vessel to the stationary well plate, the spheroids were treated with the three HDAC inhibitors (Vorinostat, Trichostatin A, and Tubacin) in concentration ranges of 0–80 µM for 48 h. After this incubation, media was changed in all wells, along with the addition of fresh media and 10 µL of Alamar Blue HS viability reagent and recording absorption readings after 4 h at 570 and 600 nm.

The IC_50_ values for both monolayer and spheroid cultures were computed by plotting the averaged absorbance readings versus concentration using the AAT Bioquest’s Inhibitory concentration (IC) 50 calculator web tool. IC_50_ curves were made in GraphPad Prism 8.

**Figure 3:**
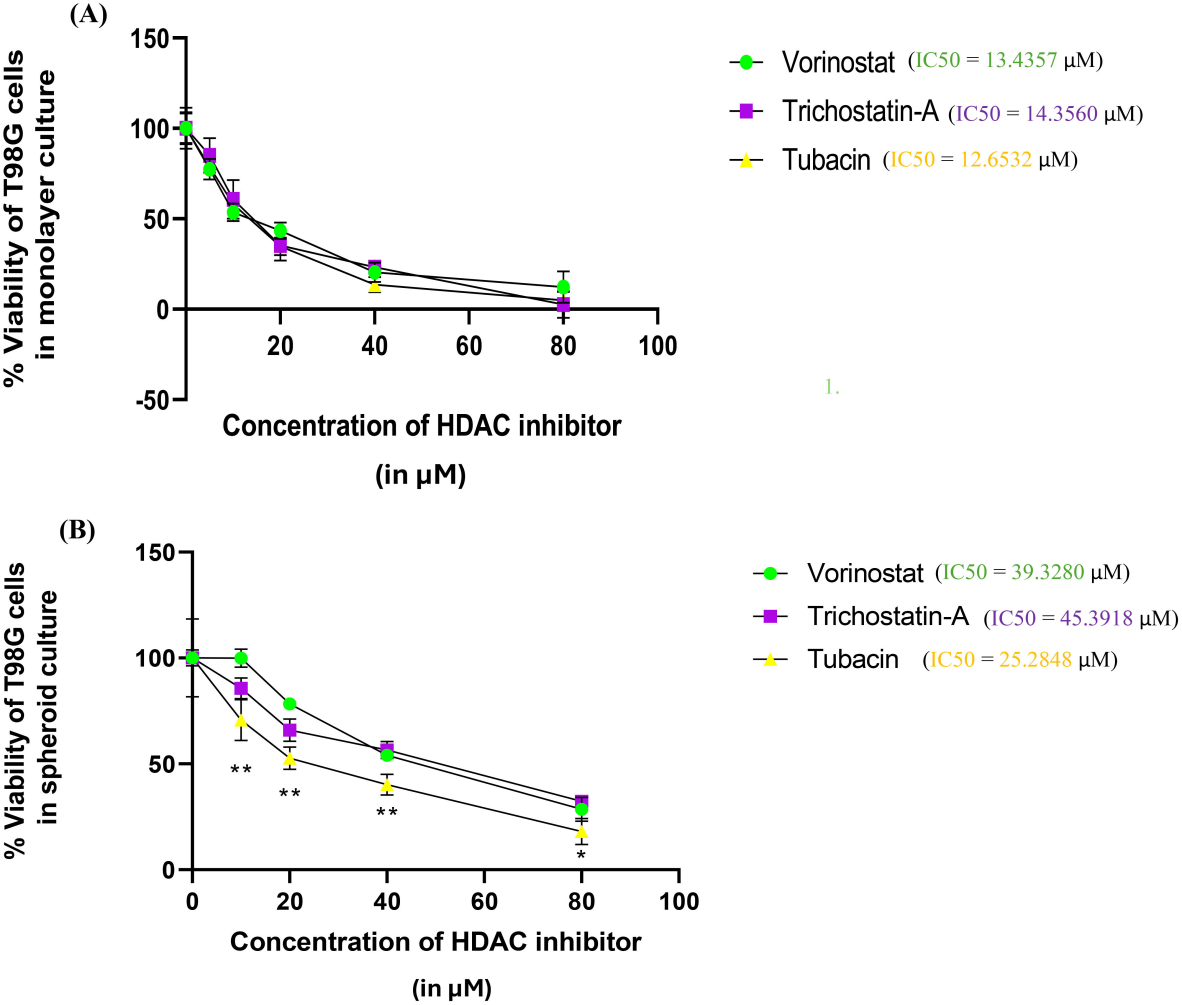
Percentage survivability versus concentration graphs for the Alamar Blue assay of the three selected HDAC inhibitors (Vorinostat, Trichostatin A, and Tubacin) on **(A)** monolayer culture and **(B)** spheroid culture of T98G cells. Graphs generated using IC_50_ calculator from the AAT Bioquest website. Statistical analysis was performed using GraphPad Prism 8. The assay was performed in triplicate. Results for % Viability for every HDAC inhibitor at each concentration were compared using one-way ANOVA with Tukey’s *post-hoc* test. In **Figure 3B**, “*” represents a *p*-value < 0.05, “**” represents a *p*-value < 0.01, and “ns” represents non-significant.

Results for % Viability for every HDAC inhibitor at each concentration were compared using one-way analysis of variance (ANOVA) with Tukey’s *post-hoc* test. The assay was performed in triplicate (**Figure 3**).

#### 2.3.1 Live–dead assay

Spheroids were treated for a period of 72 h with the IC_50_ values obtained in the 3D cultures of the three HDAC inhibitors (obtained from Alamar Blue assay)—39.32 µM for Vorinostat, 45.39 µM for Trichostatin A, and 25.28 µM for Tubacin. Spheroids in separate wells were stained with the Cyto 3D live–dead assay kit (BM01—TheWell Bioscience, Monmouth Junction, NJ). Staining reagent (2 µL) is used per 100 µL of working solution. The spheroids were incubated in the staining solution for 30 min, after which they were washed with phosphate-buffered saline (PBS) twice and then taken for imaging.

Image acquisition: Images were obtained from a Zeiss LSM 980 confocal microscope. The spheroid images were taken on a green filter for AO (excitation wavelength, 488 nm) and a red filter for PI (excitation wavelength, 514 nm) and saved as “.czi’ files. Fluorescence intensity quantitation was performed using Fiji (ImageJ) software. Each condition was taken in triplicate.

**Figure 4:**
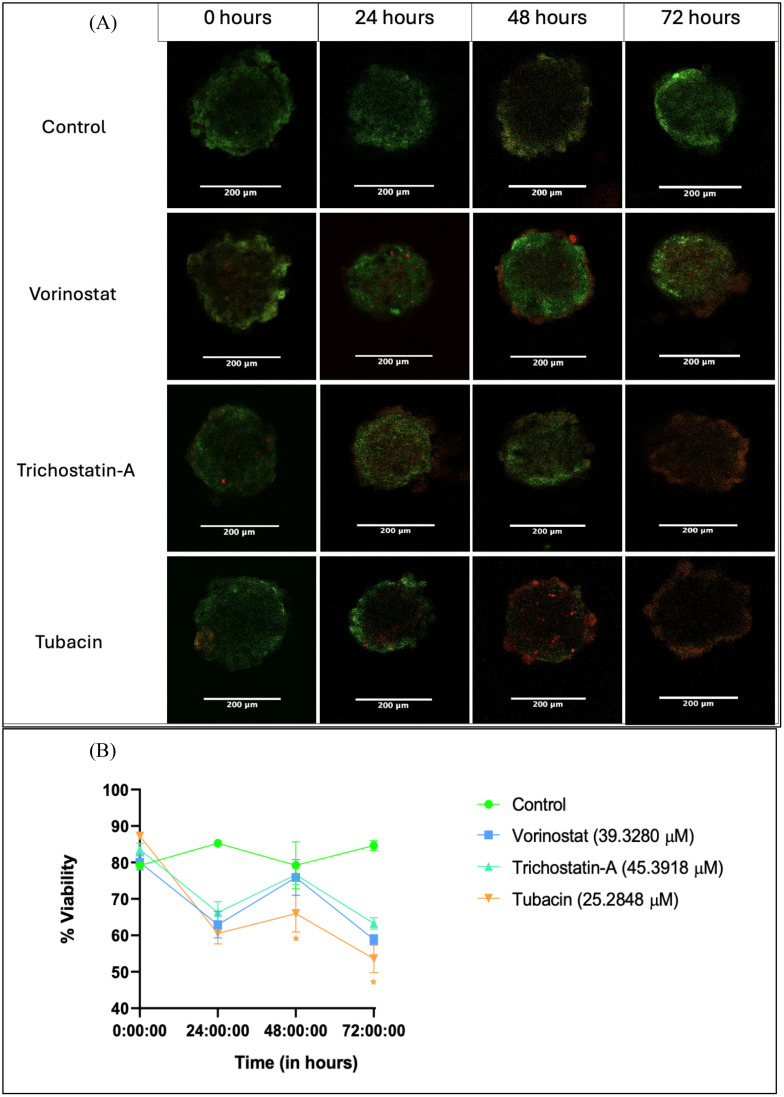
Live–dead assay for evaluation of anti-proliferative effect of HDAC inhibitors on tumor spheroids over a 72-h period. **(A)** Representative images of spheroids after 0, 24, 48, and 72 h of treatment with 3D IC_50_ concentrations of respective HDAC inhibitors. **(B)** Numerical change in the quantified intensity ratio of green dye (acridine orange) to the red dye (propidium iodide) over a time point of 0 to 72 h Images analyzed using Fiji (ImageJ) software. Statistical differences were calculated by two-way ANOVA with Tukey’s *post-hoc* test for multiple comparisons. In **Figure 4B**, “*” represents a *p*-value < 0.05 and “ns” represents non-significant.

Image analysis: Fluorescence intensity analysis was done for the images by initially converting images from both filters into grayscale images. Regions of interest (ROIs) were drawn around the stained portions and intensities were measured as “mean gray values”. The ratio of mean gray values from spheroids for GFP images to that from spheroids for DS red images was taken as a measure for viability. (For a detailed protocol, please refer to supplemental information protocol 2.) Average changes across two independent repeats are presented in **Figure 4B**. %Viability is presented as the ratio of mean gray values from live cell dye (AO) to dead cell dye (PI). Statistical differences were calculated by two-way ANOVA with Tukey’s *post-hoc* test for multiple comparisons.

### 2.4 Electric cell impedance system to measure rate of migration and late resistance in T98G cells after treatment with HDAC inhibitors

The ECIS is an instrument from Applied Biophysics (Troy, NY). The experimental setup is designed such that it measures timely fluctuations in capacitance, resistance, and impendence of each well of a microarray. The ECIS setup involves the use of arrays containing eight wells, where each well can be plated under test/control conditions (based on the experiment design). The arrays are fitted into an array station, placed within a CO_2_ incubator, connected to a Station controller (outside the incubator) via a flat wire, which is, in turn, connected to a readout device via a USB. These fluctuations are then correlated with cancer cell attachment, monolayer formation, and further changes (55).

Prior to plating the cells, all the wells of the array were filled with 600 µL of DMEM media, and a diagnostic check was performed. The procedure for this is to first insert the media-filled array in one of sample holders of the ECIS instrument at 37°C and 5% CO_2_. The array type (8W10E+ PET) was selected, and a “check” is performed to first ensure that all arrays had a good connection (indicated by the wells on the software appearing green; if any well does appears red, either remove and reattach the array and make sure media is filled all the way to the top). After the diagnostic check, a stabilizer run was performed to make sure that the electrodes are properly conditioned and ready for the experiment. Then, a standard check is performed to ensure that the current well values (for resistance, impedance, and capacitance) were in acceptable range. The array was removed from the ECIS and brought back to the biosafety cabinet.

Freshly trypsinized T98G cells were plated in five out of the eight wells of an 8W10E+ Microarray. One well was used as a media baseline. Each chamber of this array has a 600-µL capacity, and the plating conditions are described in **Table 1**. One group was kept as a regular cell control and one group was kept as a DMSO (solvent) control. The three treatments were made with a cell suspension ensuring that approximately 1 × 10^6^ cells are suspended in 600 µL of media containing the 2D IC_50_ concentration of the respective HDAC inhibitor.

**Table 1:** Treatment plating conditions for every well of an 8W10E+ array used for the ECIS experiment.

Sr no.	Group name	Conditions
1.	Media control	Only media (600 µL of DMEM media)
2.	T-98G cells (no drug treatment)	600 µL of 1 × 10^6^ cells/mL cell suspension
3.	T-98G cells (DMSO control)	600 µL of 1 × 10^6^ cells/mL cell suspension in media with 0.05% DMSO
4.	T98G + IC_50_ concentration Vorinostat	600 µL of 1 × 10^6^ cells/mL cell suspension in media with 13.44 µM Vorinostat
5.	T98G + IC_50_ concentration Trichostatin A	600 µL of 1 × 10^6^ cells/mL cell suspension in media with 14.36 µM Trichostatin A
6.	T98G + IC_50_ concentration Tubacin	600 µL of 1 × 10^6^ cells/mL cell suspension in media with 12.65 µM Tubacin

The two remaining wells on the array were filled with blank media (without any drug or cells).

After plating the conditions, the array was placed once again on the sample holder of the ECIS. Pre-run checks were performed, and the experiment was started with a run time of 168 h. For data collection and analysis, “Multiple Frequency/Time” (MFT) mode was selected for a more complete readout. For plotting the data, the readings for frequencies above 40,000 Hz are used as they are suggested to be best suited for following changes in electrical parameters due to cell spreading (55) (as at this frequency, the effect of capacitive reactance on impedance is limited).

Two readouts were calculated from the ECIS data.

#### 2.4.1 Rate of migration

This is the descriptor of overall migration from cancer cells during the initial part of the run.

The migration phase of an ECIS curve is the initial period when the resistance recorded across electrodes increases linearly with time as a result of plated cells attaching at the bottom. After a cell monolayer forms, the resistance increase over time plateaus.

For calculating ROM, we use the slope of normalized resistance in the initial roughly linear region of the resistance over time plot. Normalized resistance (*R*_norm_) is simply the ratio of resistance of condition (*R*_cond_) at time *t* to the resistance at the very beginning of the experiment (*t* = 0 h), and is mathematically given by


$$R_{\text{norm cond at time}\;t}=\;\frac{R_{\text{cond at}\;t}}{R_{\text{cond time}\;"0"}}$$

$R_{\text{cond at }t}$ is the resistance at time point *t*

$R_{\text{cond time}\;"0"}$ is the resistance at the first time point, which is 0.00068917 h.

ROM is given by


$$ROM=(\frac{R_{\text{norm cond at}\;t_{b}}\;-R_{\text{norm cond at}\;t_{a}}\;}{t_{b}-t_{a}}).[R_{\text{norm}}]$$

Where $R_{\text{norm cond at}\;t_{b}}$ is the normalized resistance at time *t_b_*

$R_{\text{norm cond at }t_{a}}\;$ is the normalized resistance at time *t_a_*

$R_{\text{norm}}$ is the slope of the initial linear increase in *R*_norm_ for all groups.

$t_{b}$ is the selected end point of period of interest. For ROM measurements, this is the time at which resistance recordings for all wells begin to plateau. (In this case, it was 18 h, the Vorinostat group took that long for plateauing while the other HDAC-inhibitor-treated groups begin to plateau at 2 h itself.)

$t_{a}$ is the selected start points of the period of interest. For ROM measurements, this is the time from the very first reading (which was 0.00068917 h^−1^ in this case) until the plateau region.

#### 2.5.2 Late resistance

This is the recorded resistance from the plateau region until the end phases of the ECIS plot.

It is a descriptor of the resultant late treatment effects on cell viability. LR for every well of the 8 W10E+ array is generally computed as


$$\bar{LR}=\frac{\sum \frac{n_{168}}{t=n_{p}}\;(R_{\text{cond}})}{(n_{168}-n_{p})\times R_{t=0}}$$

Where,

( $\bar{LR}$) is late resistance

$n_{p}$ is the reading at which plateau ends, which was reading number 388 (of 5,131)

$n_{168}$ is the last reading, which is at 168 h, that is, 7-day time point

$R_{t=0}$ is the resistance at the very start of the experiment $R_{\text{cond}}$ is the resistance of condition at time *n* (*t* → *n_p_* to *n*_168_)

A higher $\bar{LR}$ can be correlated with increased cell survival whereas lower LR values indicate reduced viability due to events like apoptosis.

**Figure 5:**
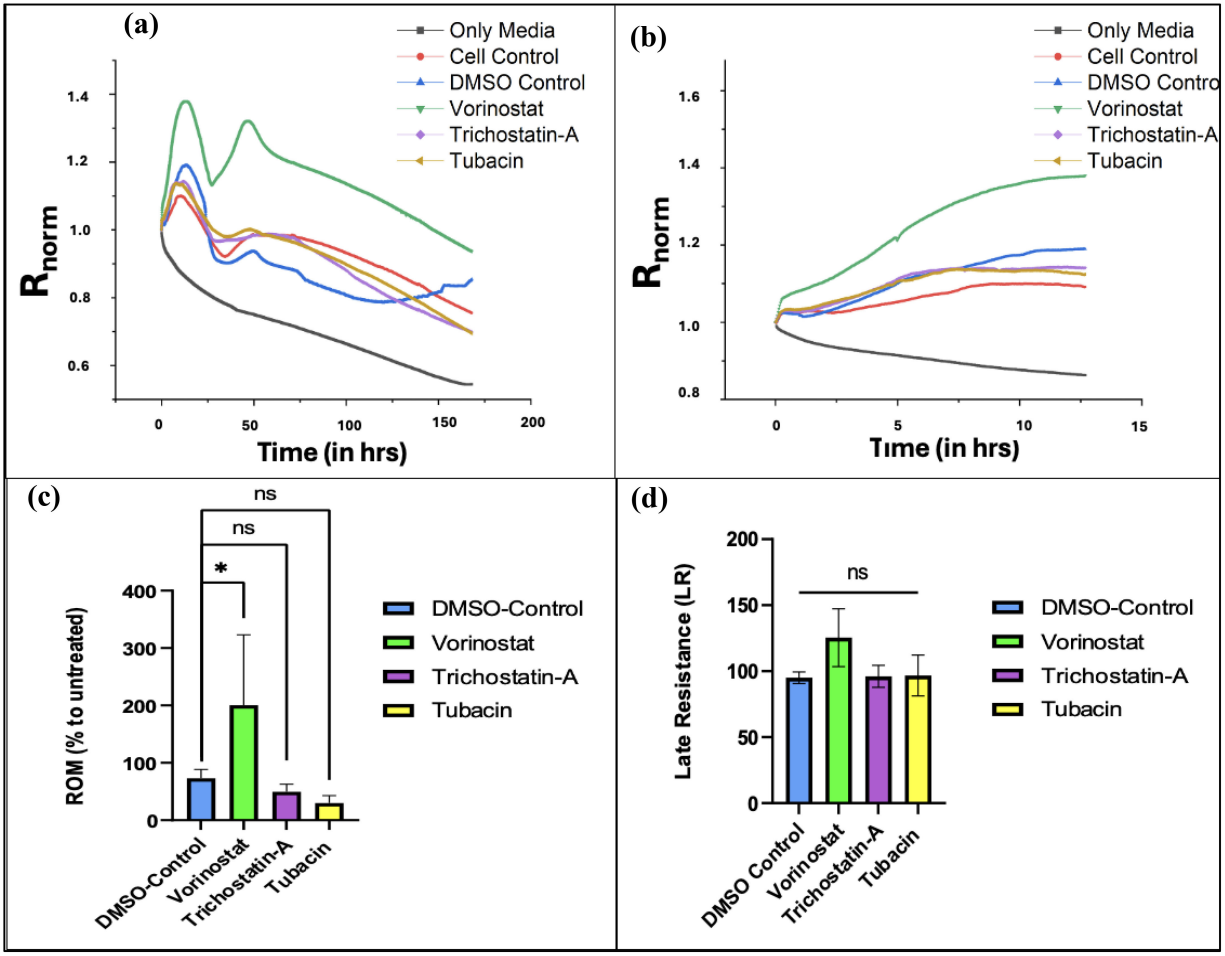
ECIS data plots. **(A)** Graph of changes in normalized resistance over time (T0 to T168) on T-98G cells plated with HDAC inhibitors. **(B)** Changes in normalized resistance from the start to plateau region. (*T*_p_ = 12.68 h). **(C)** Effect of treatments on rate of migration (ROM) from time point 0 to 12.68 h*. **(D)** Effect of treatments on late resistance ( $\bar{LR}$) from time point 12.69 to 168 h **Figures 5A and B** were made using OriginPro, **Figures 5C and D** were made using GraphPad Prism 8. Statistical analysis from averaged data set (from three independent studies) was carried out using one-way ANOVA with Bonferroni’s *post-hoc* test. *The time range is 0 to *T*_p_, where “*T*_p_” is the time point at which resistance readings stop decreasing linearly over time and begin to plateau (indicating that the cancer cells have formed a confluent monolayer at the bottom of the electrode). The experimentally recorded values for *T*_p_ were 18.02, 14.62, and 7.94 h for N1, N2, and N3 experiments (averaged 13.53 ± 5.12 h), respectively, but for the averaged data set, *T*_p_ was found to be 12.68 h.

Statistical analysis from the averaged data set (from three independent studies) was carried out using one-way ANOVA with Bonferroni’s *post-hoc* test. Results from each independent study is discussed in **Supplementary Figures 1**–**3**, while results from the averaged data set are presented in **Figure 5**.

## 3 Results

### 3.1 Cancer spheroids were successfully generated using simulated microgravity

Cancer spheroids with an average diameter of 323 µm were generated using the RCCS assembly (for the spheroid diameter calculation protocol, please refer to supplemental information protocol 1). The spheroids are allowed to grow uniformly for up to 2 weeks under regular culture conditions (37°C, 5% CO_2_, and 95% humidity), after which they were passaged to other culture vessels by initially digesting the ECM (Vitrogel) with Organoid Recovery solution and then resuspending the spheroids in fresh media, followed by mixing resuspended spheroid suspension with 2× the amount (by volume) of Vitrogel hydrogel and letting that set at room temperature in a bigger culture flask or well plate. After setting, additional media was put on to the container and the well plate was incubated in a CO_2_ incubator under standard conditions for the cell line.

### 3.2 The cytotoxicity assays yielded higher as well as distinctive IC_50_ values in spheroidal cultures compared to monolayer ones

The three selected HDAC inhibitors, Vorinostat, Trichostatin A, and Tubacin, showed comparable IC_50_ values of 13.43, 14.35, and 12.65 µM for monolayer cultures of T98G, respectively (**Figure 3A**). All the three HDAC inhibitors showed significantly higher IC_50_ values in 3D cell cultures (**Figure 3B**). The computed IC_50_ values for Vorinostat, Trichostatin A, and Tubacin for spheroidal cultures were 39.33, 45.39, and 25.29 µM, respectively.

This increase in IC_50_ concentrations is likely due to the resistive effect imparted by the 3D arrangement of cells, which leads to cells in the outermost layers of the spheroids having significantly more exposure to the cytotoxic agent than the cells in the spheroid core. This finding is in line with other researchers’ findings of drug sensitivity from 2D cultures not translating to that in 3D cultures (16, 56, 57). 3D cell culture models are considered superior for neurotoxicity screening. Additionally, the specific HDAC-6 isoform inhibitor Tubacin showed significantly higher toxicity than the non-specific HDAC inhibitors in 3D cancer spheroids. This finding is surprising since pan-HDAC inhibitors are overall considered to be more potent in their tumor-inhibiting action compared to specific inhibitors (42). For GBM, only non-specific HDAC inhibitors are currently in phase I clinical trials (41, 58).

### 3.3 Tubacin (a specific HDAC-6 inhibitor) shows greater reduction in cell viability compared to pan-inhibitors within spheroids in the 72-h live–dead assay study

We tested changes in fluorescence from AO-PI staining. Images were collected from DS red and GFP filters. The % ratio of green fluorescence signal (given by live-cell dye AO) to red fluorescence signal (given by dead-cell dye PI) was considered as a measure for viability.

Spheroid images were collected at 24, 48, and 72 h after a single dose treatment with the 3D IC_50_ concentration of each HDAC inhibitor (**Figure 4A**). The three HDAC inhibitors produced a similar decrease in % G/R ratio at 24 h, followed by a surprising increase at 48 h and then another decrease (possibly due to the long-term effect of the HDAC inhibitor) (**Figure 4B**).

Tubacin-treated groups showed significantly lower viability at the 48-h (63.98%) and 72-h (53.57%) time points, indicating the higher potency of the compound (**Figure 4B**). These results are interesting in that they differ from what was observed in the Alamar Blue assay. At 48 h, none of the HDAC-inhibitor-treated groups had viability below 50% (which should have been the case if the 3D IC_50_ values obtained were absolute). However, cytotoxicity assays are known to produce variant results based on experimental variables such as cell density-dependent proliferation, effect of vehicle, and tumor heterogenicity, among others (59).

Another reason for the non-linear result translation between assays could be the differences in end-point parameter assessed. The Alamar Blue assay measures viability based on metabolic activity due to the conversion of the dye Resazurin into the colorimetric product, Resorufin. The live–dead assay directly looks at fluorescence from the amount of nuclei positive for alive cells (for AO) vs. dead cells (for PI) (60).

### 3.4 Vorinostat had a non-specific increase in ROM whereas the other two HDAC inhibitors reduced it

We found that T98G cells treated with an IC_50_ concentration (13.43 µM) of Vorinostat had greater attachment and faster growth in the initial phase of the ECIS run. **Figure 5A** shows the experimental resistance value over time plots for all the conditions from averaged out readings from three independently performed experiments. **Table 1** describes how each condition was plated (two wells of the microarray were unused for the experiments).

For calculating ROM, we use changes in normalized resistance from the start of the experiment to the last reading after which the net linear increase begins to plateau. The slope of these curves is used to compute ROM. In our experiments, we found that the normalized resistance readings plateaued at approximately 12.67 ± 5.12 h (for more details on the results of each experiment, please refer to **Supplementary Figures 1**–**3**).

**Figure 5B** shows an increase in normalized resistance over the initial migratory period of the cells. Since the stock solutions of the three HDAC inhibitors were made in DMSO, an organic solvent (due to their lipophilic nature), a DMSO control was used (61, 62). After computing the ROM with respect to untreated cell conditions for the vehicle control along with the three treatment groups and comparing, we found that Vorinostat significantly increased the migration of T98G cells compared to other groups in the first half of the experiment (**Figure 5C**).

Vorinostat also had the highest LR parameter with respect to untreated cells, with it crossing 100%, indicating that its treatment promoted cell survival in the later stages. The other two HDAC inhibitors did reduce it below 100%, but had non-significant reduction compared to vehicle (DMSO) control.

Despite Vorinostat and Trichostatin A being pan-HDAC inhibitors, they both show variant effects on ROM and LR. This can be explained by the different degree to which both pan-HDAC inhibitors affect various intracellular targets implicated during GBM.

Vorinostat primarily works by increasing acetylation of targets like tubulin and HSP-90, whereas the known targets for Trichostatin A are GATA-4 (antitumor transcription factor) and gelsolin (actin-binding protein) (63, 64). Vorinostat has higher specificities for HDAC1 and HDAC6, while Trichostatin A has more broad-spectrum inhibition. Trichostatin A had the highest IC_50_ (indicating lower potency) as well as a slight reduction in ROM and LR. The specific HDAC-6 inhibitor Tubacin showed the most reduction in ROM compared to vehicle control; however, it also did not significantly reduce LR in the long term (see **Figure 5D**). All of these changes indicate potential inadequacies of both specific HDAC-6 and pan-inhibitors for being used as monotherapies in GBM.

## 4 Discussion

### 4.1 Comparative potency and viability effects

GBM remains among the most aggressive CNS malignancy with a highly heterogenous clinical presentation as well as no definitive cure (65). Epigenetic strategies like HDAC inhibition have drawn considerable interest for GBM due to its promise for modulating tumor plasticity, reducing relapse, or sensitizing therapy-resistant subpopulations.

In the present study, we initially determined the potency of three HDAC inhibitors under preclinical investigation, using the Alamar Blue assay. This was followed by the estimation of toxicity in spheroids using the live–dead assay and the assessment of the effect on metastasis using ECIS.

All three inhibitors showed relatively high IC_50_ values (12.5–15 µM) in monolayer conditions, reinforcing the point that current agents are not optimally potent (i.e., above the ~10 µM threshold, often considered desirable for further anticancer lead development) (66). Among them, Tubacin, the HDAC-6 selective inhibitor, showed the highest toxicity to T98G cells. Specific HDAC inhibitors being more potent than pan-HDAC inhibitors is a finding not consistently reported against all cancer cell types (67, 68). In the context of GBM, HDAC-6 has been reported to be upregulated (69, 70) and confer chemotherapy resistance in GBM cells (71, 72). This explains why specific HDAC-6 inhibitors could be more potent against GBM proliferation (compared to pan-inhibitors); however, this has to be confirmed with more HDAC-6 inhibitors.

The spheroidal cultures showed higher IC_50_ values than the monolayer ones for all three HDAC inhibitors. This finding is expected and well-documented across various studies (73, 74). The three HDAC inhibitors had more distinctive IC_50_ values from the 3D assay, indicating that our tumor spheroid culture may be ideal for comparative neurotoxicity screening for anti-GBM leads for the T98G cell line (see **Figure 3B**).

Tubacin showed higher potency in the live–dead assay as well; however, none of the three HDAC inhibitors were able to increase the fluorescence of the dead cell dye sufficiently enough to lower the % G/R fluorescence. AO-PI staining is widely used to characterize the effects of drug treatments on spheroids or 3D structures, but its results do not always translate with cytotoxicity assays such as Alamar Blue as the latter measures the mitochondrial enzyme activities rather than just the number of dead and viable cells. Tubacin indicating higher potency and cytotoxicity via the Alamar Blue and live–dead assays and at the same time not showing robust suppression of migration and long-term resistance in the ECIS assay reveals that while Tubacin may be more cytotoxic, it does not reliably outperform the pan-HDAC inhibitors (Vorinostat and Trichostatin A).

### 4.2 Migration, ECIS, and challenges of selectivity

The ECIS studies indicated interesting readouts. Even though no direct studies have been previously done to evaluate the effect of Vorinostat on the migration of T98G cells, Vorinostat has been shown to reduce migration in other glioma cell lines like U87MG at low concentrations (75) (the IC_50_ values on U87MG cells and the murine cell line GL261 were 9.7 and 6.3 µM, respectively). The increase in migration observed during our experiments (see **Figure 5C**) due to IC_50_ can be explained due to the difference in cell line and assay used for studying migration. Similarly, Trichostatin A has also previously been reported to have an inhibitory effect on T98G cells (44). It has shown an inhibitory effect on other GBM cell lines (76, 77), but in the current study, it did not significantly reduce migration compared to vehicle (DMSO control) and also did not reduce LR towards the end of our ECIS study.

For Tubacin, there were no previous studies that checked its direct use on GBM cell lines. However, another more potent HDAC-6 inhibitor, Tubastatin-A, has previously been demonstrated to reduce migration in a wound healing assay for T98G cells (51). In another study, siRNA-induced silencing of HDAC6 demonstrated a reduction in migration in T98G cells (69).

All three HDAC inhibitors demonstrated mixed or minimal anti-migration effects under the tested conditions. This contrasts with findings from studies using alternative HDAC6 inhibitors like Tubastatin-A (51, 69). This could partly be due to the nature of the migration assay.

Traditional wound healing/scratch assays involve plating cells on ECM like Matrigel and culturing them until a monolayer is formed. Then, a mechanical scratch is made to disrupt the monolayer, and the time required by the cells to form a monolayer by proliferating closer is recorded as a measure of migration. They require time-point-dependent imaging and are more mechanical (55). Similarly, transwell assays involve the measurement of direct cell migration/invasion events across permeable membranes. Cancer cells are seeded in the upper chambers of a transwell insert (generally coated with ECM) with migration in the bottom chamber (with a chemoattractant molecule), and migration is measured via end-point cell counting (via staining cells in the bottom chamber). These assays provide a simple and straightforward measure of migration but come with the disadvantage of being reliant on end-point imaging and susceptible to technical artifacts (like membrane clogging/uneven cell seeding) (78). In contrast, ECIS records real-time changes in electrical impedance via cell barrier formation, disruption (due to drug treatment/spontaneous apoptotic events) and migration (55). It may have a greater reproducibility due to a more automated measure, but it also comes with the disadvantage of difficulty visualizing single cells and being not amenable to 3D cultures (79).

It should be noted that, in the past studies, migration inhibition was not uniformly potent across all doses or cell contexts, and often was most effective when HDAC6 inhibition was combined with chemotherapy (51, 69). This contrast between cytotoxic and migratory outcomes underscored by our data emphasizes the critical need for multidimensional phenotypic screening—viability alone is not enough, especially in the context of invasion, migration, and long-term resistance.

## 5 Limitations of our study

Single-cell line model: We conducted our experiments solely in the T98G line, which, although widely used, does not reflect the spectrum of genotypes or phenotypes found in human GBM. Clinical tumors often comprise mixed populations of glioma stem cells and differentiated tumor cells. Future work should extend to primary GBM cultures, patient-derived cell lines, or the cancer stem cell-enriched population (80).

Short window for the live–dead assay: The live–dead assay was limited to a 72-h post-treatment window. Extending beyond 72 h involves media change or re-dosing, and we did not examine multi-dose, long-term visibility dynamics.

ECIS method constraints: ECIS is optimized typically for adherent GBM cell lines. Its interpretation in lines with more suspension or loose adhesion (e.g., U251 and SNB19) is more nuanced and not straightforward (54, 79). We have not yet applied ECIS to spheroid-derived migration in T98G due to interpretative complexity.

## 6 Future directions and perspectives

Targeting other HDAC isoforms: We plan to test inhibitors against other HDAC isoforms (e.g., HDAC4 or HDAC8) using the viability and migration platforms established here (81, 82).

Organoid/assembloid models: We also aim to develop cerebral organoids from iPSCs or neural progenitor cells, implant GBM cells, and study invasion and treatment effects in a brain-like microenvironment (83). Assembloids combining GBM spheroids with brain organoids can be used to model invasion dynamics (84), and co-cultures of GBM spheroids with organoids of other tissues (e.g., lung and liver) may permit an *in vitro* study of metastatic invasion and the effect of HDAC inhibitors (85).

Combinational/dual-target therapies: We aim to test dual-target inhibitors using the workflows described like CM-414, which targets HDAC-6 and phosphodiesterase-5 (PDE-5) (86) or complement axis inhibitors (87).

Repeated dosing assays: Future experiments with extended live–dead imaging time points and viability measures over longer durations are also being developed.

## 7 Conclusion

In summary, we present a rigorous *in vitro* workflow that integrates 2D monolayer assays, 3D spheroid cultures (including ECM-embedded spheroids), live–dead imaging, and ECIS-based migration assays. This multi-pronged approach allows the evaluation of newer molecular leads like HDAC inhibitors not only for cytotoxic potency but also for functional traits relevant to GBM aggressiveness: migration (ROM) and long-term survival (LR) under stress.

Our findings are mixed: while Tubacin (HDAC6-selective) outperformed pan-HDAC inhibitors with respect to reducing cell viability (lower IC_50_ values in both 2D and 3D, and greater toxicity in live–dead imaging), it failed to consistently reduce migration or LR in ECIS assays. The non-specific inhibitor Vorinostat increased migration in the beginning of the ECIS experiment. Together, our findings suggest that more potent and possibly more selective HDAC6 inhibitors—or perhaps compounds targeting additional complementary pathways—may be required to achieve meaningful anti-GBM effects. The 3D spheroid-ECM embedded model, functional assays, and the ECIS-based migration assessment provide a useful platform for future screening of such next-generation agents, enabling more reliable evaluation of migration, invasion, viability, and resistance phenotypes before moving toward *in vivo* studies or clinical translation.

We hope that by applying this workflow, future research can more effectively identify molecular leads with realistic translational potential in GBM, such that they can reduce tumor burden and limit recurrence, ultimately improving patient outcomes. These findings highlight the need for the development of newer, more potent, and isoform-selective HDAC inhibitors (or dual-target inhibitors) that can deliver both robust cytotoxicity and suppression of migration, invasion, or chemoresistance. However, future studies are needed by an *in vivo* cancer model to realize its translational potential.

## Data Availability

The original contributions presented in the study are included in the article/**Supplementary Material**. Further inquiries can be directed to the corresponding author.
