# Supplementary material for: Isoform-specific vs. pan-histone deacetylase inhibition as approaches for countering glioblastoma: an in vitro study
Source: Front Oncol. 2025 Nov 27;15:1695552. doi: 10.3389/fonc.2025.1695552 (PMC12695619; doi:10.3389/fonc.2025.1695552)
Supplement: Supplementary file 1 [file DataSheet1.docx]

Supplementary information sheet

**Protocol 1: spheroid diameter determination**:

1. Take brightfield image of spheroid from microscopes with scalebars. We used an EVOS XL core microscope with imaging system. Make sure you turn the scalebar option on the image.
2. Open the image on Fiji software.
3. In the menu, select the *straight*, segmented or freehand lines, or arrows function – ‘
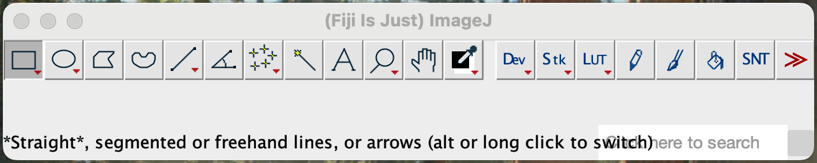
’.


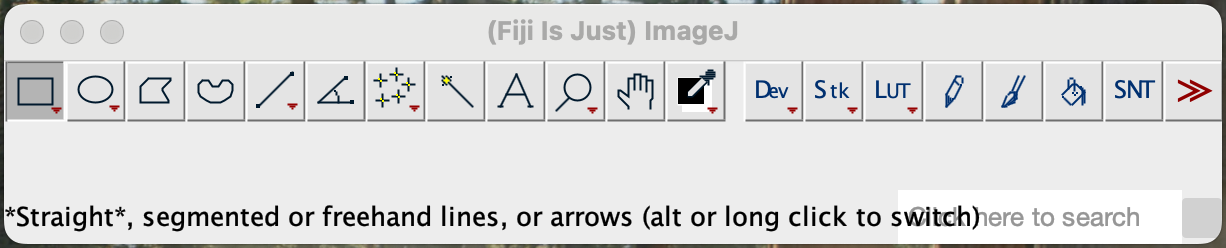


1. Draw out a horizontal line along the scale bar.
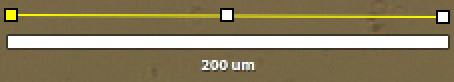

2. Go to

Analyze → Set Scale. Set the ‘unit of length’ – Micrometer and enter the known distance. Keep the pixel aspect ratio as 1. Then click ‘ok’


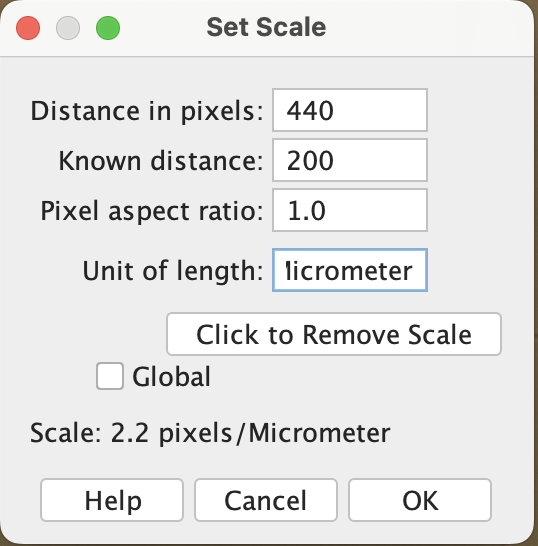


1. Using the ‘
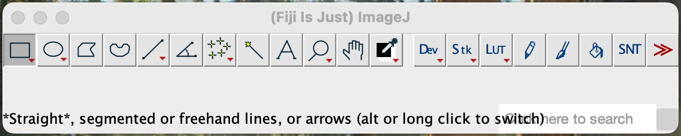
’ function, draw a line around the spheroid’s diameter and then click ‘Ctrl + M’ or ‘Command + M’
2. Fiji generates a result table with the ‘length’ column reporting diameter in the micrometer scale. (you need images with scale bars for this).

**Protocol 2: Fluorescence intensity measurement**

1. Images were recorded as ‘.czi files’ on Zeiss LSM 980 confocal fluorescence microscope.
2. Images for the green and red filters were analyzed using the mean grey value function in Fiji.
3. Next, go to Image→Lookup Tables→ Grays to convert images from both filters into greyscale images (for more accurate intensity measurement).
4. Go to Analyze→ Set Measurements , to select all the readouts you want. This is a standard example of the parameters selected by us.


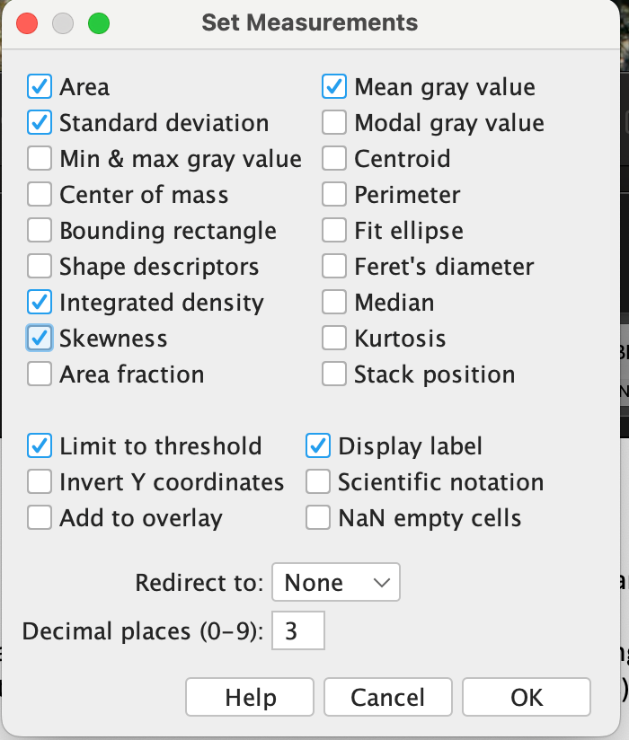


1. Click ‘ok’ and then go to image.
2. Draw regions of interest (ROI) roughly surrounding the spheroids were demarcated and put on ROI manager (by pressing ‘T’ or ‘Command + T’).
3. Finally, for measuring intensity, click ’Command + M’. Fiji generates a table with all indicated measurements.
4. Analyze the intensity from all groups. Analyze a minimum of 3 images per timepoint per group.


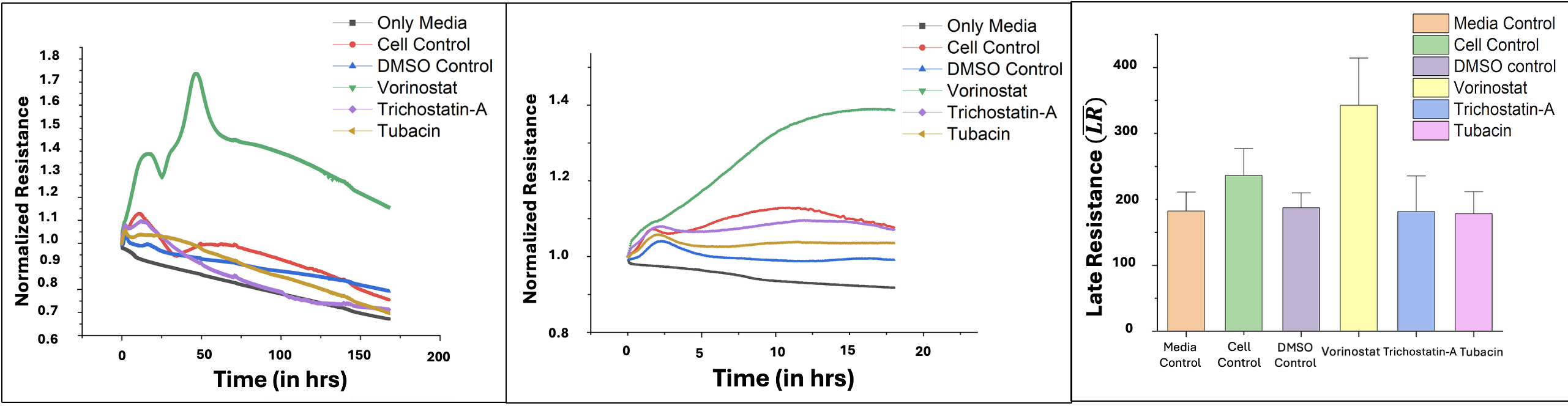


**Figure 1: ECIS N1 experiment data. (a) Normalized resistance readings for treatments plated for 168 hours, (b) R_norm_ changes from t_0_ to t_plateau_ region, and (c) bar graphs for late resistance.**

For the first experiment, Vorinostat gave the highest ROM readings as well as Late Resistance $(\bar{LR})$ paramerter. The vorinostat-treated T-98G cell groups also showed two distinct phases of migration, one from 0 to 18.02 hours and the other starting at 25.023 hr and plateauing roughly around 46.54-hour timepoint.


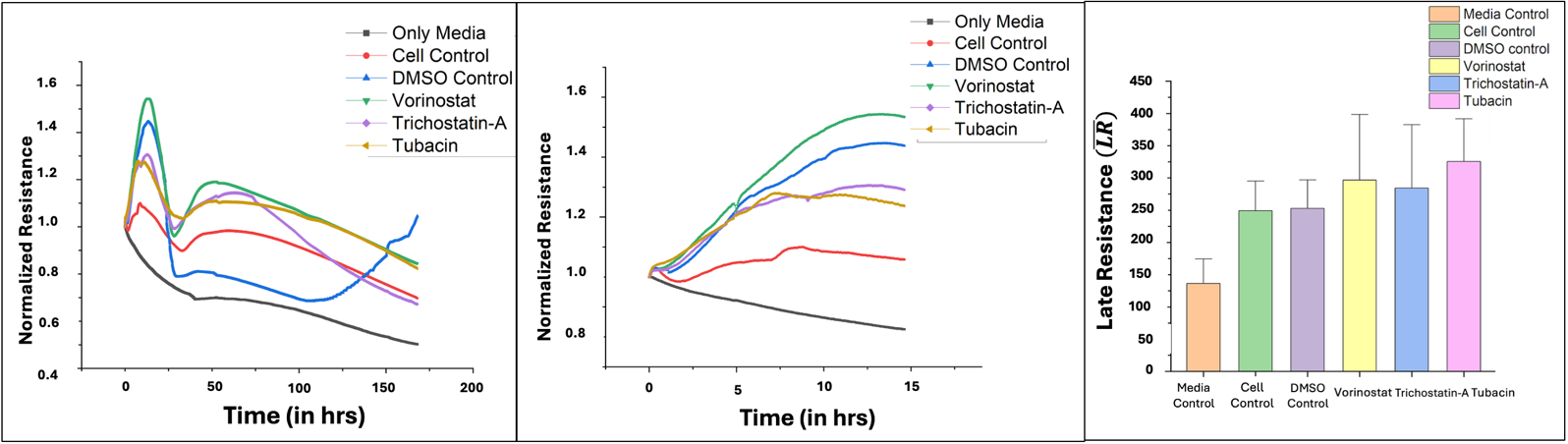


**Figure 2: ECIS N2 experiment data. (a) Resistance readings for treatments plated for 168 hours, (b) R_norm_ changes from t_0_ to t_plateau_ region, and (c) bar graphs for** $\boldsymbol{LR}$**.**

For the second experiment, the IC50 concentration of Vorinostat once again showed biphasic migration, but this time, the other pan-HDAC inhibitor (Trichostatin-A) trend and the specific HDAC-6 inhibitor, Tubacin, also showed similar trends with Tubacin having highest migration in the second phase. Towards the end of the experiment, the DMSO control had the highest migration. The late resistance of all HDAC-inhibitor treated groups was comparable with DMSO control.


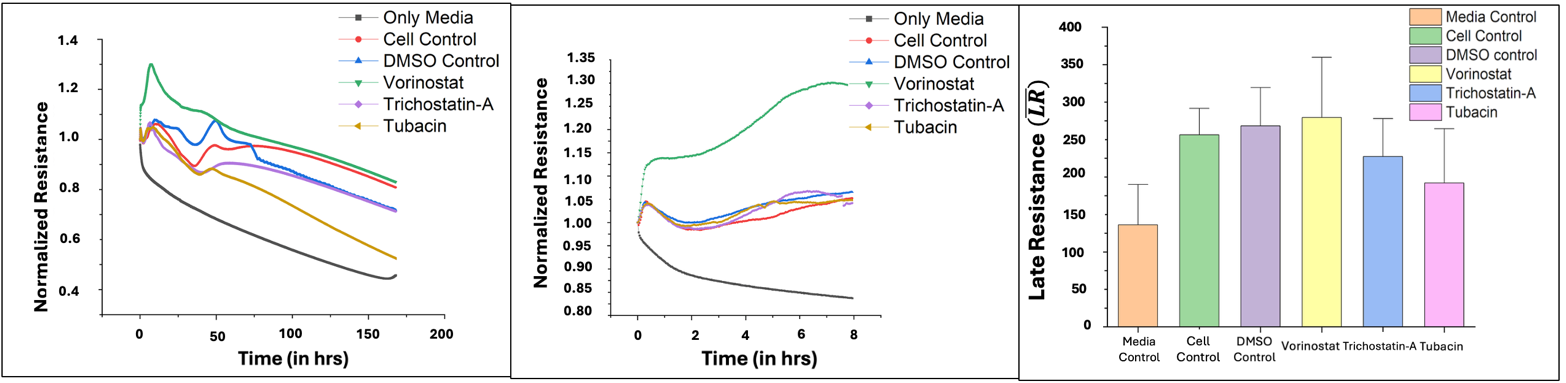


**Figure 3: ECIS N3 experiment data. (a) Normalized resistance readings for treatments plated for 168 hours, (b) R_norm_ changes from t_0_ to t_plateau_ region, (c) bar graphs for** $\bar{\boldsymbol{LR}}$**.**

During the 3^rd^ trial DMSO control and Trichostatin groups had two distinct cell migration phases. Vorinostat once again had the highest rate of migration and late resistance. The other pan-HDAC inhibitor (Trichostatin-A), and the specific HDAC-6 inhibitor (Tubacin) did reduce migration and late resistance but not to a statistically significant extent.
